# Improving Docking Performance Using Negative Image-Based Rescoring

**DOI:** 10.3389/fphar.2018.00260

**Published:** 2018-03-26

**Authors:** Sami T. Kurkinen, Sanna Niinivehmas, Mira Ahinko, Sakari Lätti, Olli T. Pentikäinen, Pekka A. Postila

**Affiliations:** ^1^Department of Biological and Environmental Science and Nanoscience Center, University of Jyvaskyla, Jyväskylä, Finland; ^2^Institute of Biomedicine, Integrative Physiology and Pharmacy, University of Turku, Turku, Finland

**Keywords:** molecular docking, docking rescoring, negative image-based rescoring (R-NiB), benchmarking, consensus scoring

## Abstract

Despite the large computational costs of molecular docking, the default scoring functions are often unable to recognize the active hits from the inactive molecules in large-scale virtual screening experiments. Thus, even though a correct binding pose might be sampled during the docking, the active compound or its biologically relevant pose is not necessarily given high enough score to arouse the attention. Various rescoring and post-processing approaches have emerged for improving the docking performance. Here, it is shown that the very early enrichment (number of actives scored higher than 1% of the highest ranked decoys) can be improved on average 2.5-fold or even 8.7-fold by comparing the docking-based ligand conformers directly against the target protein's cavity shape and electrostatics. The similarity comparison of the conformers is performed without geometry optimization against the negative image of the target protein's ligand-binding cavity using the negative image-based (NIB) screening protocol. The viability of the NIB rescoring or the R-NiB, pioneered in this study, was tested with 11 target proteins using benchmark libraries. By focusing on the shape/electrostatics complementarity of the ligand-receptor association, the R-NiB is able to improve the early enrichment of docking essentially without adding to the computing cost. By implementing consensus scoring, in which the R-NiB and the original docking scoring are weighted for optimal outcome, the early enrichment is improved to a level that facilitates effective drug discovery. Moreover, the use of equal weight from the original docking scoring and the R-NiB scoring improves the yield in most cases.

## Introduction

Molecular docking is an *in silico* technique that samples potential binding poses of ligands flexibly against the ligand-binding cavities of receptor protein structures. This ability to mimic ligand-receptor recognition at the atom level can yield valuable insight on complex and experimentally difficult to approach phenomena such as enzyme reaction mechanics or ligand-receptor association especially when it is coupled to atomistic simulations.

The main interest for docking comes from its use in computer-aided drug discovery and virtual screening experiments that aim to discover novel drug compounds from vast compound libraries—a process that ideally lowers the amount of costly experimental testing. On the one hand, the docking algorithms reproduce experimentally verified ligand binding geometries with remarkable accuracy (Kitchen et al., 2004; Warren et al., 2006; Kolb and Irwin, 2009; Meng et al., 2011). On the other hand, anybody who has used docking on routine basis can confirm that these successes are case-specific and the methodology often fails to produce sufficient enrichment (Ferrara et al., 2004; Mohan et al., 2005; Sousa et al., 2006; McGaughey et al., 2007; Plewczynski et al., 2011). In part, this hit-or-miss nature of docking is caused by the lack of relevant 3D structure data on the target proteins (Schapira et al., 2003) or inadequacies of the ligand conformer sampling (Sastry et al., 2013), but the other fundamental problem is the failure in scoring the sampled docking solutions (Wang et al., 2003; Warren et al., 2006; Plewczynski et al., 2011; Pagadala et al., 2017).

In other words, although the conformational space of the ligand binding might be sampled exhaustively, the best binding poses or the most potent compounds are not necessarily put to the top of the ranking lists by the default scoring functions (Wang et al., 2003; Ferrara et al., 2004; Cross et al., 2009; Plewczynski et al., 2011). An experienced researcher might be able to select the best pose out of 10 different conformers, but the situation becomes quickly unattainable when dealing with hundreds or thousands of compounds. The docking scoring functions put a certain weight on the specific ligand-receptor interactions such as hydrogen bonding, halogen bonding and π-π stacking but also the internal energies of the ligand conformers are considered. Despite the undeniable merits, these binding favorability or energy assessments do not always work (Chen et al., 2006; Cross et al., 2009), which means that the best pose or, more relevantly, the active compound is frequently ignored in the docking screening.

The docking solutions can be rescored after the fact to increase the yield. This is done by reassessing the favorability of the solutions utilizing a set of empirical binding descriptors that put weight on different binding characteristics. In the consensus scoring, a set of different scoring functions are employed and together they produce better enrichment than any of the functions accomplish alone (Charifson et al., 1999; Clark et al., 2002; Oda et al., 2006). Tasking more than one scoring methodology should in theory cover all the bases and, furthermore, a mix of dissimilar functions should facilitate the discovery of active hits from vast compound pools. The inherent problem with the consensus rescoring, however, is that the optimal settings are specific for each target. Accordingly, their successful use with novel targets lacking benchmark test sets is difficult to ascertain beforehand (Cheng et al., 2009).

In addition, performance enhancement might be produced by docking the ligands with different software to improve the sampling (Houston and Walkinshaw, 2013) or by optimizing and estimating the binding poses using the Poisson–Boltzmann or generalized Born and surface area continuum solvation (MM/PBSA or MM/GBSA), free energy perturbation (FEP) or solvated interaction energy (SIE) calculations (Bash et al., 1987; Kollman et al., 2000; Onufriev et al., 2004; Naïm et al., 2007; Guimarães and Cardozo, 2008; Sulea et al., 2011, 2012; Genheden and Ryde, 2015; Virtanen et al., 2015; Juvonen et al., 2016). Because these post-processing steps require a lot of extra computing, it limits their applicability in the real-world screening studies involving potentially hundreds of thousands of compounds. In addition, the success-rates of the post-processing methods vary on a case-by-case basis (Virtanen et al., 2015) and, beforehand, there is no way to tell whether the extra investment will pay out. In short, there is a genuine need for reliable rescoring methodologies that do not require a lot of extra computing resources or experiment-based tinkering.

The aim of the study was to demonstrate that by focusing solely on the shape/electrostatics complementarity between the docked ligand poses and the receptor protein's ligand-binding site, the yield of the small-molecule docking could be improved.

In the negative image-based (NIB) screening (Virtanen and Pentikäinen, 2010; Niinivehmas et al., 2011, 2015), a negative image or a NIB model is generated by inverting the shape and electrostatics of a ligand-binding cavity using a specifically tailored software PANTHER (Niinivehmas et al., 2015). The resulting NIB model is used by similarity comparison algorithms such as ShaEP (Vainio et al., 2009) the same way as ligand 3D structures extracted from the X-ray crystal structures are used in the ligand-based screening. The ligand 3D conformers, used in the similarity comparison, are generated from scratch using software such as BALLOON (Vainio and Johnson, 2007); but, notably, the conformers could also originate from molecular docking sampling.

**Figure 1:**
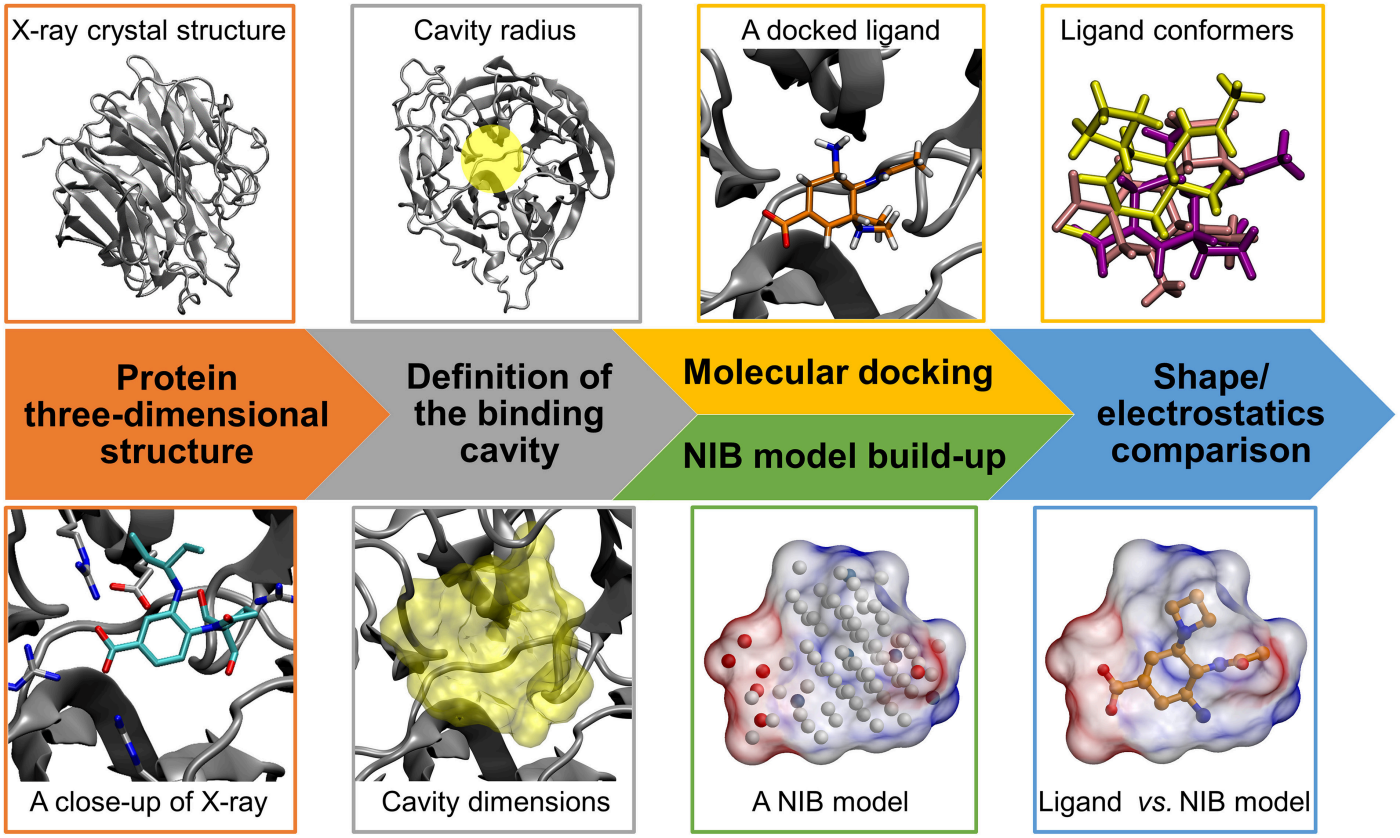
Negative image-based rescoring workflow. Firstly, the protein 3D structure (neuraminidase; gray cartoon; PDB: 1B9V) (Finley et al., 1999) and ligand 3D structures for molecular docking are prepared (e.g., protonation). Secondly, the ligand-binding cavity is outlined using a detection radius for docking (yellow transparent circle above) and NIB model generation (yellow transparent surface below). If there exist a bound ligand in the PDB entry (BANA206 as a stick model with cyan backbone in the close-up below), it can be used in defining the cavity center and/or dimensions. Thirdly, the docking of ligands into the cavity is performed using a standard docking software and multiple docking solutions or conformers are outputted for rescoring. Fourthly, a cavity-based NIB model, composed of explicit cavity points (white neutral; blue positive; red negative) is generated with PANTHER (Niinivehmas et al., 2015) for the same cavity. Fifthly, the NIB model shape/electrostatics (transparent surface with charge potential) are compared directly against the docking solutions using a similarity comparison algorithm ShaEP (Vainio et al., 2009) without geometry optimization. Those solutions matching the cavity information are given higher scores than the ones that differ.

To explore this idea further and to improve docking enrichment, the NIB screening methodology was repurposed for rescoring multiple explicit docking solutions output by the docking software PLANTS (Korb et al., 2009). The main difference between the established NIB methodology and the here introduced NIB rescoring or the R-NiB (Figure 1) is that it is performed as is. The coordinates of the cavity-based negative image and the docked ligand conformers are not superimposed or optimized for a better match. The rescoring was performed with 11 target proteins ranging from nuclear receptors such as progesterone receptor (PR) to neuraminidase (NEU) using established virtual screening benchmark libraries containing both known active and inactive decoy ligands (Huang et al., 2006; Mysinger et al., 2012). Altogether 22 different benchmark sets were used to validate the new methodology (Table 1).

**Table 1:** Target protein 3D structures used in the virtual screening.

**Target protein^a^**	**DUD**				**DUD-E**			
	**PDB code**	**Resolution (Å)**	**Ligs^b^**	**Decs^c^**	**PDB code**	**Resolution (Å)**	**Ligs^b^**	**Decs^b^**
ER-agonist	1L2I	1.95	67	2,352	–	–	–	–
ER-antagonist	3ERT	1.9	39	1,394	–	–	–	–
ER-mixed^c^	–	–	106	3,746	1SJ0	1.9	383	20,663
AR	2AO6	1.89	74	2,628	2AM9	1.64	269	14,343
GR	1M2Z	2.5	78	2,797	3BQD	2.5	258	14,986
MR	2AA2	1.95	15	535	2AA2	1.95	94	5,146
PPARγ	1FM9	2.1	81	2,906	2GTK	2.1	484	25,256
RXRα	1MVC	1.9	20	706	1MV9	1.9	131	6,935
COX2	1CX2	3.0	348	12,462	3LN1	2.4	435	23,136
PDE5	1XP0	1.79	51	1,808	1UDT	2.3	398	27,520
	1UDT^d^	2.3	–	–	1XOZ^d^	1.37	–	–
PR	1SR7	1.46	27	967	3KBA	2.0	293	15,642
NEU	–	–	–	–	1B9V	2.35	98	6,197
CYP3A4	–	–	–	–	3NXU	2.0	170	11,797

a *AR, androgen receptor; COX2, cyclo-oxygenase 2; CYP3A4, cytochrome P450 3A4; ER, estrogen receptor alpha; GR, glucocorticoid receptor; MR, mineralocorticoid receptor; NEU, neuraminidase; PPARγ, peroxisome proliferator activated receptor gamma; PR, progesterone receptor; RXRα, retinoid X receptor alpha; PDE5, phosphodiesterase type 5*.

*ER-agonist, ER-antagonist and ER-mixed refer to ligand sets containing ER-specific agonists, antagonists or both, respectively*.

b *Number of active ligands (Ligs) and decoy (Decs) molecules after preprocessing with LIGPREP*.

c *In the DUD database, ER agonists and antagonists are separated into two separate datasets, but in the case of the DUD-E the ligands are mixed. For comparison, the ER datasets in the DUD were also mixed*.

d *Used in the NIB model generation*.

As a whole, the results show that the R-NiB produces moderate or excellent early enrichment improvements using the basic settings in the NIB model generation and similarity screening. In most cases, the early enrichment of the docking can be improved also by consensus scoring, in which the original PLANTS docking scoring and the PANTHER/ShaEP-based R-NiB scoring are given an optimal weight ratio. What is more, the rescoring indicates that the hit rate is typically enhanced even when both of these scoring functions are bluntly given equal (50/50%) weight in the consensus scoring.

In summary, the success of the R-NiB approach in sorting out the active ligands from the inactive molecules is directly related to the fact that the shape/electrostatics complementarity between the ligand and the receptor is an essential part of the complex formation.

## Materials and methods

### Ligand set preparation

The ligand sets, including the active and inactive decoy compounds, were acquired from the DUD (A Directory of Useful Decoys) (Huang et al., 2006) and DUD-E (A Database of Useful (Docking) Decoys -Enhanced) (Mysinger et al., 2012) databases for the target proteins (Table 1). The initial 3D coordinates for the DUD ligands were converted to the SMILES (Simplified Molecular-Input Line-Entry System) format using STRUCTCONVERT in MAESTRO 2017-1 (Schrödinger, LLC, New York, NY, USA, 2017). LIGPREP in MAESTRO was used to generate OPLS3 charges and tautomeric states for both the DUD and DUD-E ligand sets at pH 7.4. Next, both of the ligand sets were converted to the SYBYL MOL2 format using MOL2CONVERT in MAESTRO. The back-and-forth conversion between MOL2 and SMILES formats was done with the DUD ligands to avoid potential bias of the original 3D conformations for the molecular docking (Zoete et al., 2016).

### Protein preparation

The 3D structures of the target proteins, which were used in the molecular docking and the NIB model generation, were acquired from the Protein Data Bank (PDB) (Berman et al., 2000; Burley et al., 2017). All of the used PDB entries are listed in Table 1. The benchmarking was done mainly using the PDB entries listed for the DUD and DUD-E datasets and, thus, both the docking and rescoring could work better or worse using different structures. The necessary PDB entry editing (Figure 1) such as the removal of bound ligands from the active sites was done in the BODIL Molecular Modeling Environment (Lehtonen et al., 2004). The protein residues were protonated with the default settings in REDUCE3.24 (Word et al., 1999). The X-ray crystal structure waters were left in the deprotonated state for NIB model building.

### Molecular docking

The molecular docking of the DUD and DUD-E compound sets (Figure 1) into the ligand-binding sites of the target proteins was performed using PLANTS1.2 (Korb et al., 2009). The default settings were used in the docking screenings. Accordingly, the initial docking scoring was performed with the ChemPLP that combines the PLP (Piecewise Linear Potential) with GOLD's Chemscore (Korb et al., 2009). The centroid coordinates of ligands bound in the target protein structures were used as the binding site centers in the docking. A relatively large binding site radius of 10 Å was generally used in the docking. The radius was slightly reduced for glucocorticoid receptor (GR; 9 Å) based on the size of the ligand-binding site. Altogether 10 docking solutions were output for each compound for the purpose of NIB rescoring. The idea is to provide enough different docking solutions for the rescoring.

### Negative image-based model generation

The negative images or the NIB models of the target proteins' ligand-binding cavities (Figure 1) were prepared using the default settings in PANTHER0.18.15 (Niinivehmas et al., 2015). The centroids used in the NIB model generation were based on the centroid coordinates of the ligand compounds bound in the original protein 3D structures the same way as was done with the docking. The NIB models were prepared in three different ways: (1) the NIB model size and dimensions were adjusted using the box radius option (6–10 Å); (2) the cavity size was limited to a certain radius (1.5–3.0 Å) from the bound ligand in the original structure using the ligand distance limit option; (3) when available and producing better results, a model (referred as PANTHER model) was taken also from a prior NIB screening study (Niinivehmas et al., 2015). The NIB model coordinates for all new NIB models are included in the Supplementary Material.

### Negative image-based rescoring

The NIB rescoring (or the R-NiB; Figure 1) of the original docking solutions was performed using ShaEP1.0.7.915 (Vainio et al., 2009). The shape and electrostatics of each docking solution was compared directly against the template NIB models without superimposing or optimizing their coordinates (–noOptimization option). Both the shape and electrostatics were given equal amount of weight (ESP = 0.5) in the ShaEP similarity scoring (default option). Because altogether 10 conformers were outputted for each docked compound, even those solutions given lower scores by PLANTS (Korb et al., 2009) could be later considered in the PANTHER/ShaEP-based (Virtanen and Pentikäinen, 2010; Niinivehmas et al., 2011, 2015) NIB rescoring.

### Rescoring with alternative methodologies

The docking poses initially scored by PLANTS using ChemPLP scoring function were also rescored using an alternative scoring function PLP in PLANTS. Otherwise, default options were used in the PLANTS-based rescoring. In addition, the docking solutions were also re-ranked using the default settings of XSCORE1.2.1 (Wang et al., 2002) for comparison. The XSCORE has three empirical scoring functions HPSCORE, HMSCORE and HSSCORE that can be fine-tuned on case-by-case basis to improve the docking yield. None of the scoring functions produced markedly better early enrichment separately for the docking results at least without special adjustments; thus, the software's default option of using X-CSCORE consensus scoring with all three functions was utilized.

### Consensus scoring

The R-NiB relies heavily on the initial success of the docking software used to generate the multiple docking poses for the rescoring phase, because no coordinate optimization or extra sampling is performed (Figure 1). Essentially, this means that the used PLANTS scoring is intrinsically influencing the R-NiB yield in this study. The consensus scoring takes this aspect further by directly incorporating the initial ChemPLP docking scoring with the R-NiB scoring. All possible combinations, in which both PLANTS- and ShaEP-based scoring were given different weights, were considered with 5% interval and those consensus scoring settings producing the highest early enrichment are discussed. The scores for each docked conformer outputted by PLANTS and ShaEP were normalized to fit into the scale from 1 to 0 and then combined for a consensus score.

### Table and figure preparation

Figures 1, **4**, **5** were prepared using BODIL (Lehtonen et al., 2004), MOLSCRIPT2.1.2 (Kraulis, 1991), RASTER3D3.0.2 (Merritt and Murphy, 1994), and VMD1.9.2 (Humphrey et al., 1996). The area under curve (AUC) values (Tables 2, 3), the early enrichment values (Tables 4, 5) were calculated with ROCKER0.1.4 (Lätti et al., 2016). The enrichment factors were calculated as true positive rate when 1 or 5% of the decoy molecules have been found (EFn%_DEC_; see equation below) in order to make future comparison reliable against other methodologies (Lätti et al., 2016).

(1) $${\text{EF}}_{\text{n}\%\text{DEC}}=\frac{{\text{Ligs}}_{\text{n}\%\text{DEC}}}{{\text{Ligs}}_{\text{all}}}\times 100$$

**Table 2:** The AUC values for the DUD datasets.

**Docking**		**Rescoring**				
**Target protein**	**PLANTS ChemPLP**	**R-NiB: Ligand distance^a^**	**R-NiB: Box radius^b^**	**R-NiB: prior models^c^**	**XSCORE**	**PLANTS PLP**
ER-agonist	0.81 ± 0.03	0.78 ± 0.03	0.76 ± 0.03	0.79 ± 0.03	0.82 ± 0.03	0.78 ± 0.03
ER-antagonist	0.81 ± 0.04	0.85 ± 0.04	0.77 ± 0.04	0.82 ± 0.04	0.71 ± 0.05	0.83 ± 0.04
ER-mixed	0.64 ± 0.03	**0.77** ± **0.03**	**0.70** ± **0.03**	**0.74** ± **0.03**	0.66 ± 0.03	0.61 ± 0.03
AR	0.80 ± 0.03	**0.84** ± **0.03**	0.81 ± 0.03	–	0.79 ± 0.03	0.78 ± 0.03
GR	0.60 ± 0.03	**0.80** ± **0.03**	**0.83** ± **0.03**	**0.84** ± **0.03**	**0.75** ± **0.03**	0.53 ± 0.03
MR	0.80 ± 0.07	**0.93** ± **0.05**	**0.91** ± **0.05**	0.82 ± 0.07	**0.92** ± **0.05**	0.78 ± 0.07
PPARγ	0.95 ± 0.02	0.92 ± 0.02	0.87 ± 0.03	–	0.81 ± 0.03	0.94 ± 0.02
PR	0.63 ± 0.06	0.52 ± 0.06	0.50 ± 0.06	0.50 ± 0.06	0.51 ± 0.06	0.58 ± 0.06
RXRα	0.78 ± 0.06	**0.89** ± **0.05**	0.84 ± 0.06	**0.90** ± **0.05**	**0.97** ± **0.02**	0.76 ± 0.06
COX2	0.81 ± 0.01	**0.93** ± **0.01**	**0.92** ± **0.01**	**0.95** ± **0.01**	0.65 ± 0.02	**0.85** ± **0.01**
PDE5	0.71 ± 0.04	0.67 ± 0.04	0.67 ± 0.04	0.72 ± 0.04	0.54 ± 0.04	0.66 ± 0.04

*If the rescoring produced higher AUC value in comparison to the initial docking (no overlapping standard error ranges), those numbers are shown in bold*.

a *The ligand distance limit used in PANTHER varied between the targets due to the size/shape differences of the binding cavities and the screened ligand sets. Limits included 1.5 Å (ER, AR, MR, PPARγ, PR RXRα, and COX2), 2.0 Å (GR), and 3.0 Å (PDE5)*.

b *The box radius varied between the targets due to the size/shape differences of the binding cavities and screened ligand sets. The radiuses included 6.0 Å (GR, PR and COX2), 7.0 Å (ER-mixed, MR and RXRα), and 8.0 Å (ER-agonist, ER-antagonist, AR, PPARγ and PDE5)*.

c *The previously published PANTHER models, optimized for regular NIB screening, were taken from a prior study (Niinivehmas et al., 2015)*.

**Table 3:** The AUC values for the DUD-E datasets.

**Docking**		**Rescoring**				
**Target protein**	**PLANTS ChemPLP**	**R-NiB: Ligand distance^a^**	**R-NiB: Box radius^b^**	**R-NiB: Prior models^c^**	**XSCORE**	**PLANTS PLP**
ER-mixed	0.74 ± 0.01	0.66 ± 0.02	0.65 ± 0.02	–	0.71 ± 0.01	0.70 ± 0.02
AR	0.54 ± 0.02	**0.76** ± **0.02**	**0.73** ± **0.02**	**0.75** ± **0.02**	**0.65** ± **0.02**	0.53 ± 0.02
GR	0.54 ± 0.02	**0.74** ± **0.02**	**0.76** ± **0.02**	**0.70** ± **0.02**	**0.69** ± **0.02**	0.51 ± 0.02
MR	0.55 ± 0.03	**0.74** ± **0.03**	**0.76** ± **0.03**	**0.68** ± **0.03**	**0.69** ± **0.03**	0.53 ± 0.03
PPARγ	0.85 ± 0.01	0.77 ± 0.01	0.75 ± 0.01	–	0.66 ± 0.01	0.84 ± 0.01
PR	0.63 ± 0.02	**0.74** ± **0.02**	**0.75** ± **0.02**	0.63 ± 0.02	**0.67** ± **0.02**	0.61 ± 0.02
RXRα	0.77 ± 0.02	**0.83** ± **0.02**	**0.81** ± **0.02**	**0.81** ± **0.02**	**0.85** ± **0.02**	0.70 ± 0.03
COX2	0.66 ± 0.01	**0.75** ± **0.01**	0.65 ± 0.01	–	0.62 ± 0.01	0.67 ± 0.01
PDE5	0.78 ± 0.01	0.72 ± 0.02	0.70 ± 0.02	–	0.58 ± 0.02	0.74 ± 0.01
NEU	0.85 ± 0.02	**0.89** ± **0.02**	**0.89** ± **0.02**	–	0.68 ± 0.03	0.56 ± 0.03
CYP3A4	0.61 ± 0.02	0.60 ± 0.02	0.60 ± 0.02	–	0.53 ± 0.02	0.60 ± 0.02

*If the rescoring produced higher AUC value in comparison to the initial docking (no overlapping standard error ranges), those numbers are shown in bold*.

a *The ligand distance limit used in PANTHER varied between the targets due to the size/shape differences of the binding cavities and screened ligand sets. Limits included 1.5 Å (ER-mixed, AR, PPARγ, PR, and COX2), 2.0 Å (MR, RXRα, NEU, PDE5, and CYP3A4) and 3.0 Å (GR)*.

b *The box radius varied between the targets due to the size/shape differences of the binding cavities and screened ligand sets. The radiuses included 6.0 Å (AR, GR, MR, COX2, NEU, and PR), 7.0 Å (PDE5, RXRα, and CYP3A4) and 9.0 Å (PPARγ) and 10.0 Å (ER-mixed)*.

c *The previously published PANTHER models, optimized for regular NIB screening, were taken from a prior study (Niinivehmas et al., 2015)*.

**Table 4:** The enrichment given as true positive rates for the DUD datasets.

**Docking**			**Rescoring**				
**Target protein**	**EF %_DEC_**	**PLANTS ChemPLP**	**R-NiB: ligand distance^a^**	**R-NiB: box radius^b^**	**R-NiB: prior models^c^**	**XSCORE**	**PLANTS PLP**
ER-agonist	1%	17.9	**37.3**	**31.3**	23.9	19.4	10.4
	5%	44.8	52.2	58.2	59.7	52.2	26.9
ER-antagonist	1%	15.4	**28.2**	7.7	12.8	15.4	12.8
	5%	33.3	43.6	25.6	38.5	25.6	35.9
ER-mixed	1%	0.0	**11.3**	**1.9**	**2.8**	**2.8**	0.0
	5%	20.8	23.6	5.7	8.5	6.6	7.5
AR	1%	17.6	27.0	12.2	–	9.5	14.9
	5%	40.5	45.9	45.9	–	31.1	39.2
GR	1%	6.4	**11.5**	**16.7**	**12.8**	29.5	3.8
	5%	15.4	**28.2**	**28.2**	**29.5**	50.0	14.1
MR	1%	26.7	33.3	13.3	0.0	0.0	33.3
	5%	60.0	73.3	40.0	26.7	40.0	60.0
PPARγ	1%	69.1	79.0	22.2	–	21.0	66.7
	5%	84.0	86.4	65.4	–	48.1	85.2
PR	1%	3.7	**33.3**	**33.3**	**29.6**	**18.5**	3.7
	5%	11.1	**40.7**	**40.7**	**40.7**	**22.2**	7.4
RXRα	1%	5.0	**35.0**	**20.0**	**20.0**	**70.0**	0.0
	5%	30.0	**80.0**	**45.0**	**80.0**	**85.0**	30.0
COX2	1%	13.5	**43.7**	**40.5**	**62.6**	9.2	**20.1**
	5%	35.3	**70.4**	**64.1**	**83.0**	20.1	44.8
PDE5	1%	13.7	**31.4**	**31.4**	13.7	3.9	9.8
	5%	25.5	**37.3**	**39.2**	23.5	5.9	25.5

*Those EF%_DEC_ values that are at least 1.5-fold compared to the initial docking are shown in bold*.

a *The ligand distance limit used in PANTHER varied between the targets due to the size/shape differences of the binding cavities and the screened ligand sets. Limits included 1.5 Å (ER-agonist, ER-mixed, AR, MR, PPARγ, RXRα, and COX2) and 2.0 Å (GR and PR), 3.0 Å (ER-antagonist) and 4.0 Å (PDE5)*.

b *The box radius varied between the targets due to the size/shape differences of the binding cavities and screened ligand sets. The radiuses included 6.0 Å (MR and COX2), 7.0 Å (AR and PR) and 8.0 Å (ER's, GR, PPARγ and RXRα) and 9.0 Å (PDE5)*.

c *The previously published PANTHER models, optimized for regular NIB screening, were taken from a prior study (Niinivehmas et al., 2015)*.

**Table 5:** The enrichment given as true positive rates for the DUD-E datasets.

**Docking**			**Rescoring**				
**Target protein**	**EF%_DEC_**	**PLANTS ChemPLP**	**R-NiB: ligand distance^a^**	**R-NiB: box radius^b^**	**R-NIB: prior models^c^**	**XSCORE**	**PLANTS PLP**
ER-mixed	1%	21.7	18.3	5.5	–	6.3	12.8
	5%	36.6	32.6	20.1	–	24.8	28.7
AR	1%	1.5	**13.0**	**5.6**	**8.9**	1.9	0.4
	5%	7.1	**23.0**	**15.2**	**22.3**	7.8	5.2
GR	1%	1.2	**4.7**	**3.5**	**5.8**	1.2	1.2
	5%	12.0	**22.5**	12.8	**17.4**	10.5	10.1
MR	1%	3.2	**11.7**	**6.4**	3.2	1.1	1.1
	5%	19.1	25.5	19.1	18.1	8.5	11.7
PPARγ	1%	24.2	4.5	10.3	–	5.0	19.6
	5%	57.0	24.4	32.4	–	13.8	48.3
PR	1%	2.0	**4.4**	**3.8**	**3.8**	2.0	2.4
	5%	17.1	17.1	11.6	17.4	11.6	15.0
RXRα	1%	11.5	6.9	1.5	10.7	15.3	1.5
	5%	37.4	25.2	12.2	23.9	45.8	19.8
COX2	1%	5.7	2.3	0.5	–	2.1	**9.9**
	5%	21.6	19.1	4.1	–	6.4	25.1
PDE5	1%	11.3	10.6	3.8	–	1.5	8.8
	5%	28.1	25.9	14.1	–	7.0	24.4
NEU	1%	4.1	**13.3**	**6.1**	–	1.0	0.0
	5%	32.7	42.9	35.7	–	4.1	4.1
CYP3A4	1%	7.1	7.6	5.3	–	2.4	6.5
	5%	12.9	**18.8**	15.3	–	6.5	13.5

*Those EF%_DEC_ values that are at least 1.5-fold compared to the initial docking are shown in bold*.

a *The ligand distance limit used in PANTHER varied between the targets due to the size/shape differences of the binding cavities and screened ligand sets. Limits included 1.5 Å (ER-mixed, AR, PDE5, GR, MR, PR and COX2), 2.0 Å (RXRα, NEU and CYP3A4) and 3.0 Å (PPARγ)*.

b *The box radius varied between the targets due to the size/shape differences of the binding cavities and screened ligand sets. The radiuses included 6.0 Å (AR, GR, MR and NEU), 7.0 Å (RXRα, PR, PDE5 and CYP3A4), 8.0 Å (COX2), 9.0 Å (PPARγ) and 11.0 Å (ER-mixed)*.

c *The previously published PANTHER models, optimized for regular NIB screening, were taken from a prior study (Niinivehmas et al., 2015)*.

**Figure 2:**
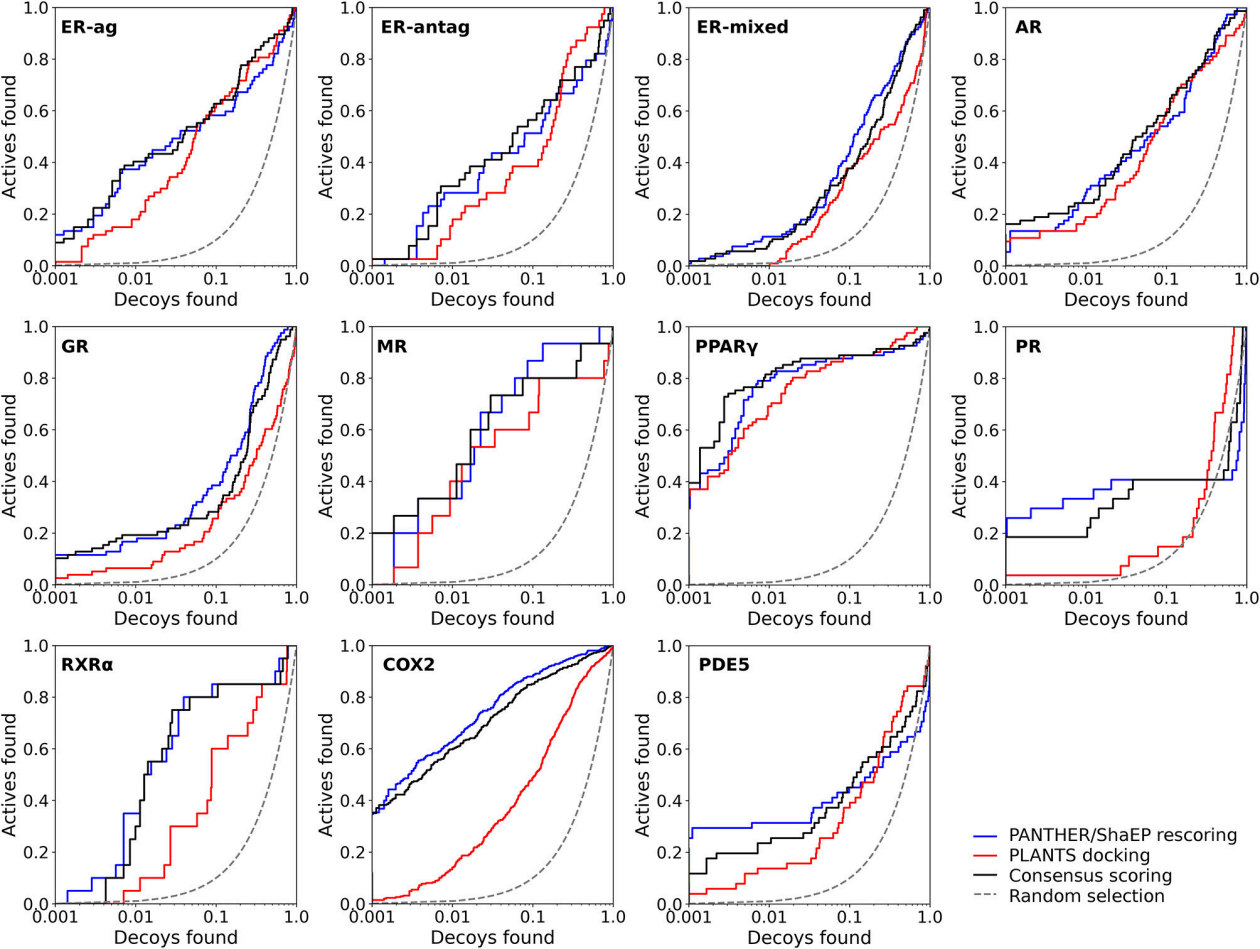
The semi-logarithmic receiver operating characteristics plots for the docking and negative image-based rescoring with the DUD dataset. Only those R-NiB results with the highest early enrichment were plotted (EF1%_DEC_ in Table 5). The red line shows the original docking enrichment by PLANTS, the blue line gives the result after PANTHER/ShaEP-based rescoring, and the black line gives the result from consensus scoring where both of them are given equal weight (50/50%). The dashed line outlines the random selection (AUC = 0.50). The semi-log10 scale is used only for the *x* axis to highlight the very early enrichment or lack thereof.

**Figure 3:**
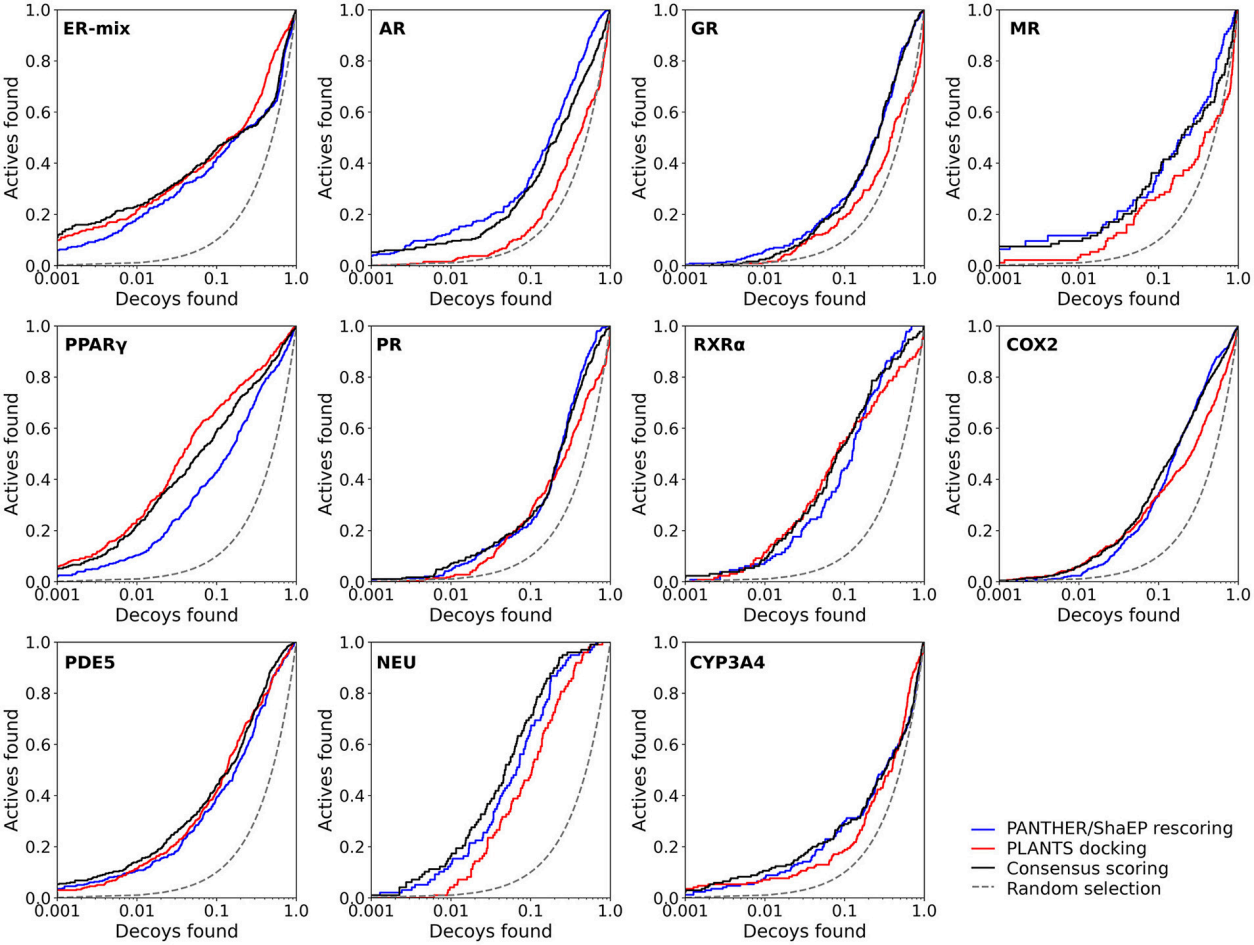
The semi-logarithmic receiver operating characteristics plots for the docking and negative image-based rescoring with the DUD-E dataset. Only those R-NiB results with the highest very early enrichment were plotted (EF1%_DEC_ in Table 6). With retinoid X receptor alpha (RXRα), the results are shown for the model (ligand exclusion of 2.0 Å; Table 6) producing the highest very early enrichment, which is visible in the plotted curve. For interpretation see Figure 2.

In Equation (1), Ligs_n%DEC_ is the number of ligands ranked higher than n % of the decoys whereas Ligs_all_ is the total number of all ligands in the dataset. The receiver operating characteristics (ROC) curves were plotted using ROCKER with the semi-log10 scale (only x axis logarithmic) in Figures 2, 3 to highlight the very early enrichment of the actives. The standard deviation for the AUC is acquired in ROCKER utilizing the derived error for the Wilcoxon statistic (Hanley and McNeil, 1982). The Wilcoxon statistic estimates the probability of ranking a random ligand higher than a random decoy, which is equivalent to the value of AUC; thus, making the errors also equal.

## Results

### Negative image-based rescoring of docking solutions

The aim of the negative image-based rescoring or R-NiB (Figure 1) is to rescore existing molecular docking solutions and, by doing so, enrich active hits from a vast pool of compounds. The enrichment is achieved by comparing the shape/electrostatics similarity between the ligand conformers and the negative image of the target protein's ligand-binding cavity. The established NIB methodology (Virtanen and Pentikäinen, 2010; Niinivehmas et al., 2011, 2015) is employed in building the cavity-based NIB models of the target proteins' ligand-binding sites (PANTHER) and in comparing them against each docking solution (ShaEP). The starting point of the R-NiB workflow (Figure 1) is that the ligands are docked into the same target protein's cavity using a standard docking algorithm and, preferably, multiple solutions that roughly fit into the cavity are outputted for the rescoring.

### Molecular docking produces moderate or high enrichment in the benchmarking

The AUC and early enrichment values (Tables 2, 3) show that the molecular docking, performed with PLANTS (Korb et al., 2009), worked relatively well with both the DUD and DUD-E datasets (Huang et al., 2006; Mysinger et al., 2012). With the DUD, the AUC values ranged from 0.60 to 0.95 indicating either moderate or substantial enrichment of actives with a majority of the targets (Tables 3). Markedly, the docking for the estrogen receptor alpha agonists (ER-agonist; AUC = 0.81), PR (AUC = 0.63) and the peroxisome proliferator activated receptor gamma (PPARγ; AUC = 0.95) worked so well that the AUC values were not improved by the R-NiB (Table 2). A side note, the DUD sets are small, containing 15–348 actives (Table 1) and, accordingly, a difference of a few active ligands in the ranking can sometimes have disproportionate effects on the AUC values. The docking worked also with the more demanding DUD-E ligand sets, containing a lot more of actives and decoys (Table 1), as the AUC values were typically well above 0.50 (Table 3). The AUC values could not be improved with the ER-mixed (AUC = 0.74), PPARγ (AUC = 0.85), phosphodiesterase type 5 (PDE5; AUC = 0.78) and cytochrome P450 3A4 (CYP3A4; AUC = 0.61) DUD-E sets using the R-NiB (Table 3).

Instead of the AUC values, it is often more practical to concentrate on the early enrichment when estimating the success of the virtual screening. That is to say, paradoxically, a high AUC value does not necessarily guarantee that the very top results contain active hits despite the fact that it is a good metric for estimating the overall success-rate of the screening. By large, the docking struggled in ranking the actives to the very top of the list, when inspecting the EF1%_DEC_ or EF5%_DEC_ values with the DUD and DUD-E datasets (Tables 4, 5). Accordingly, the very early enrichment or EF1%_DEC_ was improved by the R-NiB with all of the DUD sets (Table 4). With the DUD-E, the R-NiB could not produce improvement for the ER-mixed (EF1%_DEC_ = 21.7%), PPARγ (EF1%_DEC_ = 24.2%), retinoid X receptor alpha (RXRα; EF1%_DEC_ = 11.5%), cyclo-oxygenase 2 (COX2; EF1%_DEC_ = 5.7%), and PDE5 (EF1%_DEC_ = 11.3%; Table 5), however, in the remaining six datasets the early enrichment was improved notably (discussed below). The ROC curves, which were plotted using the semi-log10 scale to highlight the very early enrichment, corroborate the numerical trends for both of the benchmark datasets (Figures 2, 3).

### Negative image generation for rescoring is a straightforward process

The NIB model has to contain key features of the target protein's ligand-binding cavity in order to produce enrichment by the R-NiB (Figure 1). Firstly, the shape and size of the model should be limited to the cavity area that facilitates the ligand binding. Secondly, if the cavity contains vital hydrogen bond acceptor or donor groups, the NIB model must reflect those features in its charge properties. Each data point in the NIB model can be tested and adjusted iteratively using validated ligand sets that include both active and inactive compounds. This sort of “trial-and-error” refinement is generally not feasible and, accordingly, the R-NiB methodology was applied here using default easy-to-replicate PANTHER/ShaEP settings (Vainio et al., 2009; Niinivehmas et al., 2015). Effective models were acquired by simply adjusting the cavity detection box radius or by limiting the cavity dimensions with the ligand distance limit in PANTHER (Niinivehmas et al., 2015). The model generation relied solely on the PDB entry used also in the docking and generally the first-tried basic settings were enough to improve the enrichment (Tables 2–5; Figures 2, 3). For comparison, the rescoring was also performed with prior PANTHER models (Tables 2–5) optimized for the standard NIB screening (Niinivehmas et al., 2015).

### Negative image-based rescoring improves the early enrichment with most targets

The R-NiB (Figure 1) does not rely on superimposing or geometry optimization prior to the similarity comparison of the docking solutions against the cavity-based NIB models. In a nutshell, either the docked ligand poses outputted by the docking software match the cavity-based NIB models or they do not—the similarity score (from 1 to 0) of ShaEP reflects this reality. Therefore, it is crucial that the initial docking has sampled the ligand conformers thoroughly and produces “correct” ligand poses that can be discovered by the R-NiB. Understandably, the rescoring cannot enrich active compounds, if they are docked completely outside the cavity space that was used in the NIB model generation.

With the DUD datasets (Huang et al., 2006), the AUC values from docking were improved somewhat or greatly with most of the target proteins using the R-NiB (Table 2). The AUC improvement was sizeable with the GR (0.60 vs. 0.84), RXRα (0.78 vs. 0.90), mineralocorticoid receptor (MR; 0.80 vs. 0.93) and COX2 (0.81 vs. 0.95) to name a few examples (Table 2). Moreover, the R-NiB could improve the AUC values substantially even with the more demanding DUD-E sets (Mysinger et al., 2012) where the docking scoring started to falter (Table 3). This positive effect in favor of the R-NiB was seen with a multitude of target proteins, including the androgen receptor (AR; 0.54 vs. 0.76), GR (0.54 vs. 0.74), MR, (0.55 vs. 0.74), PR (0.63 vs. 0.74), RXRα (0.77 vs. 0.83), and COX2 (0.66 vs. 0.75). The AUC values worsened or improved marginally for the CYP3A4 (0.61 vs. 0.60) and NEU (0.85 vs. 0.89), respectively, but in these cases the results remained within the margin of error (Table 3). The R-NiB clearly could not improve the AUC values for the PDE5, PPARγ and ER-mixed with the DUD-E datasets (Table 3). The PDE5 and ER-mixed datasets are particularly demanding, because they both contain two distinct ligand groups for which one cannot build a single satisfactory NIB model (Niinivehmas et al., 2011).

As stated above, it is more important that the virtual screening produces the highest possible early enrichment rather than the best AUC value. To this end, the R-NiB was able to improve the early enrichment somewhat or substantially with most of the target proteins included in the DUD datasets (Table 4). The EF1%_DEC_ improvement ranged from 1.9 to 49.1% between the different targets. On average the EF1%_DEC_ or EF5%_DEC_ improvement was 3.3-fold or 1.8-fold, respectively, but, alas, the EF1%_DEC_ of PR improved 9.0-fold using the R-NiB. A close inspection of the semi-logarithmic ROC curves (Figure 2) indicates that the very early enrichment produced by the R-NiB was always as good as or better than that of the original docking scoring (well above the random rate; Figure 2). This suggests that the rescoring generally has a positive effect for the yield with the tested DUD datasets. The EF1%_DEC_ improvement (Table 4) was most prominent with the COX2 (13.5 vs. 62.6 %), but the R-NiB worked exceptionally well also based on the EF5%_DEC_ for example with the RXRα (30.0 vs. 80.0%), COX2 (35.3 vs. 83.0%), PDE5 (25.5 vs. 39.2%) and ER-agonist (44.8 vs. 59.7%).

Based on the early enrichment values (Table 4) and the plotted ROC curves (Figure 3), the overall performance of the R-NiB with the DUD-E dataset showed similar trends as with the DUD (Table 3; Figure 2). The improvement over the original docking was on average 2.5-fold for the EF1%_DEC_ (Table 5) despite the fact that the DUD-E ligand sets are much larger than the smaller but better curated DUD datasets (Table 1). For example, the EF1%_DEC_ improvement of 2.1% (from 2.0 to 4.1%) with PR might seem minor at the first glance, but in terms of absolute compound numbers it is a marked uptick from the discovery of six to 13 actives over the original docking. The EF1%_DEC_ (Table 5) was improved by the R-NiB substantially with the AR (1.5 vs. 13.0%), MR (3.2 vs. 11.7%) and NEU (4.1 vs. 13.3%). Although in the case of the RXRα the EF1%_DEC_ values suggested that the docking scoring worked better than the R-NiB (Table 5), a close inspection of the semi-logarithmic ROC plot shows that the rescoring actually produced higher very early enrichment (EF0.5%_DEC_ 6.1 vs. 3.8%; Figure 3). The EF5%_DEC_ was improved on average 1.3-fold for these targets (Table 5) and, for example, the GR (12.0 vs. 22.5%) received a 1.9-fold improvement.

### Negative image-based rescoring is both ultrafast and efficient

For the purpose of comparison, the original docking solutions were also re-evaluated using empirical rescoring algorithm XSCORE (Wang et al., 2002) and the PLP scoring function in PLANTS. Target-specific settings for ligand-receptor interactions such as hydrogen bonding or hydrophobicity are considered via multivariate analysis in XSCORE. Although the R-NiB generally produced better enrichment than XSCORE, the latter algorithm excelled with both the DUD and DUD-E datasets for the RXRα (Tables 2–5). The rescoring with the PLP function in PLANTS could only in some cases (e.g., COX2) improve the original ChemPLP-based ranking and, generally, the R-NiB produced substantially better results (Tables 2–5).

The use of non-default XSCORE settings could have produced higher early enrichment; however, similar fine-tuning of the R-NiB models or even PLANTS settings could likely have improved the enrichment as well. By adjusting the assortment of the cavity charge points capable of hydrogen bonding and/or lowering/increasing the weight of the electrostatics in the similarity screening generally improves the enrichment. For example, in our test runs the R-NiB produced notably better early enrichment (EF1%_DEC_ 12.2–23.0%) for the DUD set of the AR with the box radius option when only a few cavity points were added or removed instead of using the default NIB model (data not shown). In fact, one could even over-emphasize certain properties (e.g., charge) artificially in the NIB model to produce better enrichment in the rescoring than what the default settings would otherwise allow. Because this kind of rescoring bias does not alter the actual ligand poses, the preferred docking solutions remain within the realm of possible. The situation can be entirely different, if the original docking scoring function, affecting the ligand conformer sampling, is altered radically; i.e., unrealistic conformations could be put forward.

Excluding the time taken for the NIB model generation, the actual rescoring performed with ShaEP is computationally very inexpensive; spending only a fraction of the time required for the initial docking. This is possible, because no ligand conformer sampling or even geometry optimization between the NIB model and docked ligand conformers is done. In fact, the ShaEP-based scoring with the DUD sets for the ER-agonist (1.94 ms/comp. vs. ~24.4 ms/comp.), PDE5 (3.81 ms/comp. vs. ~35.7 ms/comp.), and COX2 (2.43 ms/comp. vs. ~54.0 ms/comp.) was at least 10 times faster than the XSCORE rescoring, which is already very fast. Similarly, rescoring with PLP function in PLANTS took roughly double the time with the ER-agonist (1.94 ms/comp. vs. ~3.21 ms/comp.), PDE5 (3.81 ms/comp. vs. ~7.15 ms/comp.), and COX2 (2.43 ms/comp. vs. ~4.54 ms/comp.) datasets, when compared to the R-NiB. These benchmark numbers vary depending on the computer set-up. Here, the software were run using a single Intel Xeon CPU (W3670 3.2 GHz) and RAM 12 GB DDR 1333 MHz in a LINUX desktop. The absolute size of the NIB model and that of the compounds being rescored affect the R-NiB performance; however, the differences in the wall time are minor.

## Discussion

The negative image-based rescoring or the R-NiB is a truly novel way of rescoring docking solutions, because it does not rely on the use molecular mechanics force fields, empirical or knowledge-based descriptors in evaluating the favorability of the ligand binding. For example, the binding free energy is not considered in any shape or form during the rescoring. Although the selected atom charges and van der Waals radiuses affect the NIB model generation profoundly, the ShaEP-based rescoring itself is a simple matter of shape/electrostatics comparison. No force field-based sampling or even coordinate superimposition is needed. The NIB models can be trained for optimal effect using experimental ligand sets with the “trial-and-error” approach, but generally this is not needed.

### Applicability of negative image-based rescoring

**Figure 4:**
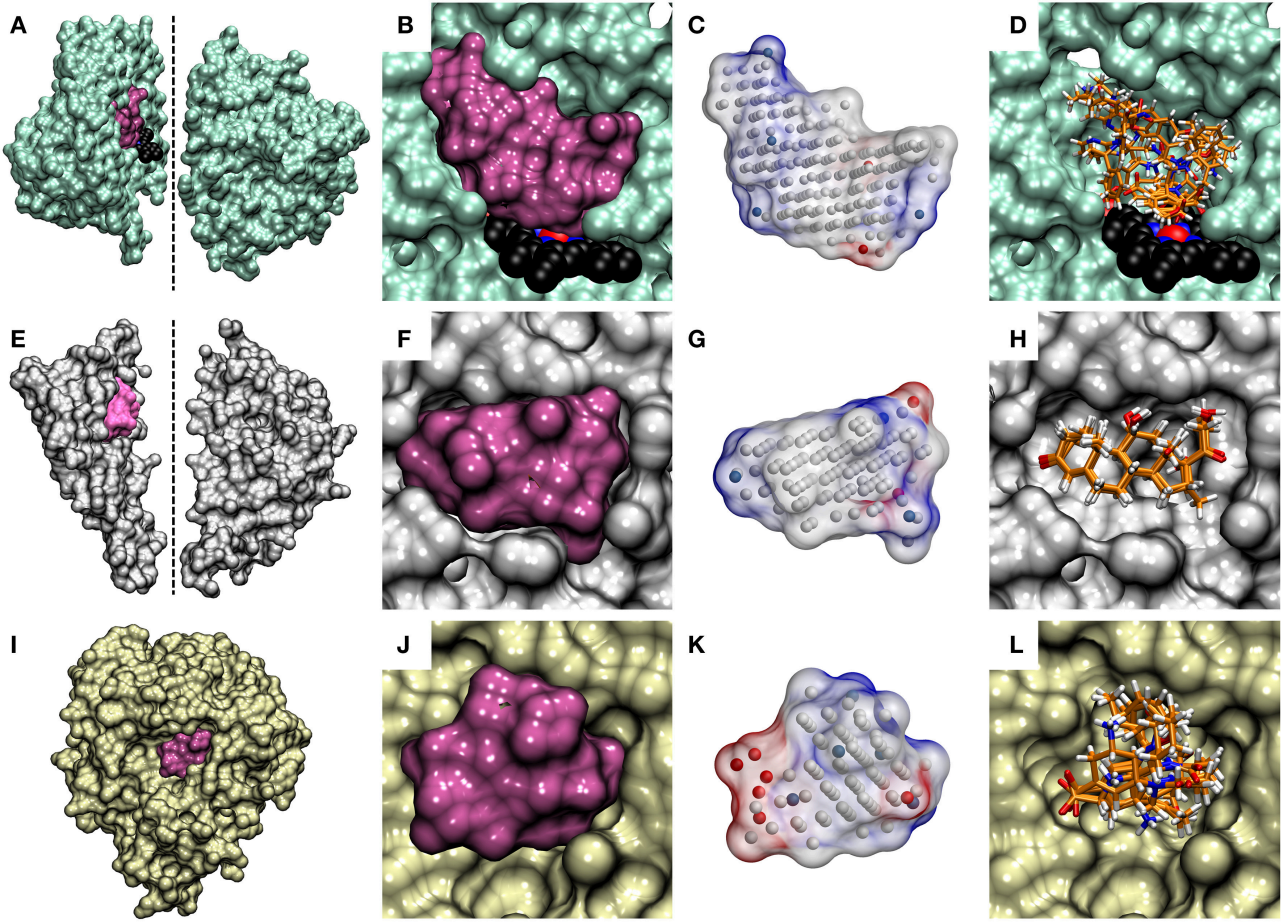
The cavity-based NIB models and the docking solutions are aligned. The protein 3D structures of **(A)** cytochrome P450 3A4 (CYP3A4; lime; PDB: 3NXU) (Sevrioukova and Poulos, 2010), **(E)** glucocorticoid receptor (GR; white; PDB: 1M2Z) (Bledsoe et al., 2005) and **(I)** neuraminidase (NEU; yellow; PDB: 1B9V) (Finley et al., 1999) are shown as opaque surfaces on the far left. With CYP3A4 and GR, the X-ray crystal structures are shown in two sections to highlight the buried locations of their active sites (mauve opaque surfaces) at the center. The dotted lines indicate the cutting planes for the cross-sections chosen for the illustration. The prosthetic heme group is shown as a CPK model (black backbone) for CYP3A4. With NEU, the enzyme's active which opens directly from the protein surface, is only partially buried and, thus, no cross-sectioning was done. The contours of the active sites of **(B,C**) CYP3A4, **(F,G)** GR, and **(J,K)** NEU are shown both as opaque surfaces and finalized NIB models (transparent surfaces with charge potential) in the cross-section close-ups. The red, blue, and white dots in the NIB model indicate the negative, positive and neutral cavity dots (or filler atoms) constituting the negative image. The docked poses of five known active compounds (stick models with orange backbone) for **(D)** CYP3A4, **(H)** GR, and **(L)** NEU from PLANTS are shown stacked in the far right.

A NIB model can be built for virtually any target protein as long as there is a solid idea where the potential small-molecule binding or initial docking should happen. The target pocket can be a well-defined and enclosed cavity (see CYP3A4 in Figures 4A–D and GR in Figures 4E–H), an opening on the protein surface (see NEU in Figures 4I–L), a sub-cavity, a groove or even a small dent on the protein surface (Figure 4). The R-NiB results with the benchmark sets confirm this hypothesis, because the method improves docking enrichment with a variety of different target proteins (Tables 2–5; Figures 2, 3) and, more importantly, with physically different kind of ligand-binding cavities (Figure 4). The enrichment values (Tables 2–4) and semi-logarithmic ROC curves (Figures 2, 3) show that the R-NiB (Figure 1) clearly improves the yield with a multitude of DUD-E datasets, including the nuclear receptors AR, GR, MR, and PR, but also with entirely different kind of target protein NEU.

Overall, the R-NiB results (Tables 2–5; Figures 2, 3) show that a satisfactory enrichment can be acquired in most cases by building NIB models by simply adjusting the cavity detection radius or by limiting the cavity search area using a receptor-bound ligand included in the PDB entry (Figures 1, 4). Having protrusions outside this cavity space do not necessarily worsen any ligand's similarity score a lot (a marginal penalty inflicted in the ShaEP scoring); however, it is important to understand that those ligand segments outside the cavity will be effectively ignored in the rescoring.

So, the emphasis of R-NiB is resolutely on the cavity's negative image (Figure 4) and it is recommended that unpractically large ligands for the cavity in question are filtered away before docking and/or rescoring. Essentially, docking sizable ligands with a lot of rotatable bonds (e.g., PPARγ datasets) or with particularly large cavities (e.g., PDE5) is likely to produce errors or difficult ascertain alternative poses that cannot be reliably rescored using the R-NiB. Despite this, in theory, the R-NiB could be used to rescore even docked peptides (not tested here) as long as their binding is dependent on the shape/electrostatics complementarity with the cavity. This narrow focus on the area designated by the NIB model for the ligand binding makes the R-NiB (Figure 1) truly a precision technique.

The downside of this narrow focus is that it also limits the usability of different benchmark test sets in evaluating the R-NiB (Figure 1). If the test set contains active compounds that bind into completely different or only partially connected ligand-binding sites in the target protein, the R-NiB cannot possibly rank all those ligands high up in the list using a single NIB model (Figure 4). Moreover, when dealing with large ligand-binding cavities such as the active site of PDE5, where inhibitors can have very different binding locations and poses, with very little overlap, and/or water molecules play a big role in coordinating the ligand binding, a single NIB model simply cannot provide all the necessary information needed for the enrichment. One can try to solve this issue by curating the ligand sets better, limiting the search radius for docking or by applying multiple NIB models to the task. Naturally, this level of focus is not a problem when working in an actual screening project, in which the efforts are centered on a specific binding site or subcavity.

### Recognizing biologically relevant ligand-binding poses

**Figure 5:**
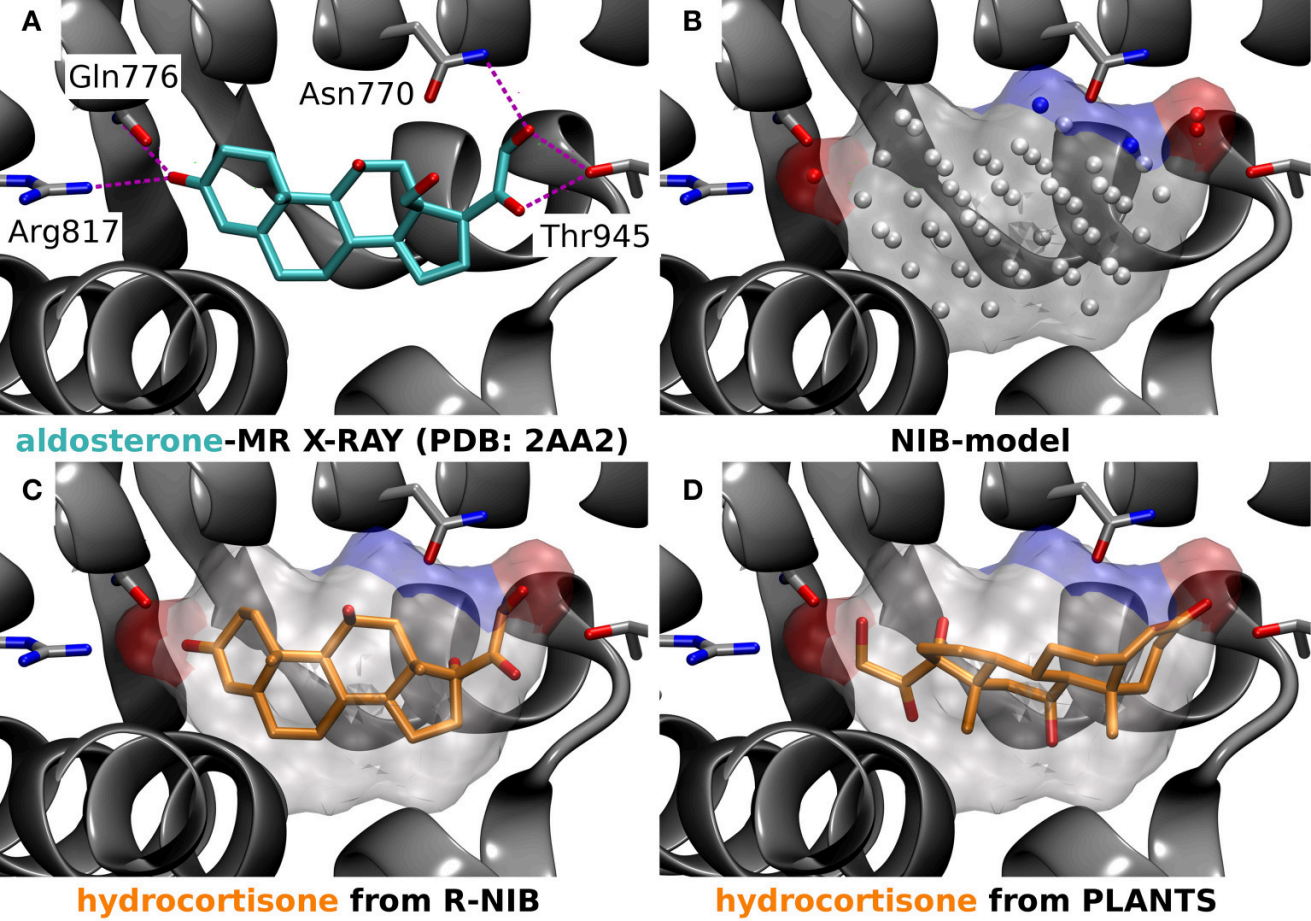
A negative image-based rescoring example with mineralocorticoid receptor. **(A)** The X-ray crystal structure of mineralocorticoid receptor (MR; silver cartoon model; PDB: 2AA2) (Bledsoe et al., 2005) and the amino acid residues (stick models) making hydrogen bonds (magenta dotted lines) with the inhibitor aldosterone (stick model with cyan backbone) are shown. **(B)** The negative image or NIB model (transparent surface) of the MR active site was build using the same PDB entry (Bledsoe et al., 2005) and the 1.5 Å ligand distance limit option in PANTHER. The red and blue dots depict the negatively and positively charged cavity points, respectively, whereas the white dots are neutral. **(C)** The rescored pose (rank #13) of hydrocortisone (stick model with orange backbone) reminds closely the experimentally verified pose of its structural analog aldosterone (**A** vs. **C**). **(D)** Hence, the pose of hydrocortisone given the highest score by PLANTS (rank #17), showing a reversed pose in comparison to the aldosterone (**A** vs. **D**), is likely erroneous **(D)**.

The R-NiB is not optimizing the ligand positioning inside the protein's ligand-binding pocket, but merely comparing the earlier produced docking poses against the cavity's shape/electrostatics (Figure 1). The highest scored poses for the active compounds might not differ from the original docking; however, the enrichment can improve due to lower ranking of the inactives by the R-NiB. In fact, improvement in the enrichment values is not an absolute guarantee that the “correct” conformers are discovered during the rescoring. With certain ligand-binding pockets and compounds it is very difficult to conclude what is the actual binding pose and there might even exist more than one valid pose (Mobley and Dill, 2009). One can attempt to address this issue by looking at the individual docking solutions, their exact binding interactions and, ultimately, compare them against the experimentally validated data for the same compound or its closely-related structural analogs (Figure 5). For example, the R-NiB seems to be able to recognize the biologically relevant binding pose of hydrocortisone with the MR whereas the original docking scoring fails (Figure 5).

Because the R-NiB can only reorder the docking solutions and if all of the ligand conformers are docked in a completely “wrong” way or even outside the ligand-binding pocket, the “correct” pose or ligand cannot emerge on top of the results list. This is true for all rescoring methodologies as they mainly reshuffle existing solutions. To a certain extent, this is the case even for force field-based post-processing methodologies, because the initial ligand-receptor complex is crucial for the sampling as well. In certain cases even a partial shape/electrostatics match with the cavity-based NIB model can give the docked compound a substantially higher ranking and improve the enrichment. By docking the decoys mostly outside the binding cavity, one could also improve the enrichment as long as the actives reside at the site. Here, it was made sure that the docked compounds and the generated NIB models occupied roughly the same 3D space in relation to the protein. The match between the cavity space and the outputted docking solutions is highlighted for the CYP3A4 (Figure 4C vs. Figure 4D), GR (Figure 4G vs. Figure 4H), and NEU (Figure 4K vs. Figure 4L) in Figure 4.

### Consensus scoring—finding the balance between the scoring functions

If the initial docking produced the “correct” or at least reasonable pose for the active compound but it was not favored by the docking software, in theory one should be able recognize it from the multiple outputted poses using a superior scoring method. In reality, all of the scoring methodologies excel on some targets and ligand sets for different and sometimes even conflicting reasons. Because both the original docking software PLANTS (Korb et al., 2009) and the similarity comparison algorithm ShaEP (Vainio et al., 2009) output their own scores for each ligand conformer, it is possible to normalize and combine the results and adjust their relative weight with different targets (Tables 6, 7).

**Table 6:** The consensus scoring of the DUD Datasets.

**Target protein**	**Optimal weight**						**Equal weight**			
	**ShaEP weight^a^**	**AUC**	**EF1%_DEC_**	**ΔEF1%_DEC_^b^**	**EF5%_DEC_**	**ΔEF5%_DEC_^b^**	**EF1%_DEC_**	**ΔEF1%_DEC_^b^**	**EF5%_DEC_**	**ΔEF5%_DEC_^b^**
ER-agonist	0.70	0.81 ± 0.03 (↔)	41.8	4.5	56.7	4.5	40.3	3.0	53.7	1.5
ER-antagonist	0.55	0.78 ± 0.04 (↓)	35.9	7.7	43.6	0.0	35.9	7.7	43.6	0.0
ER-mixed	0.90	0.77 ± 0.03 (↑)	11.3	0.0	26.4	2.8	7.5	−3.8	29.2	5.6
AR	0.25	0.85 ± 0.03 (↑)	32.4	5.4	47.3	1.4	28.4	1.4	50.0	4.1
GR	0.60	0.76 ± 0.03 (↑)	19.2	2.5	26.9	−1.3	19.2	2.5	25.6	−2.6
MR	1.0	0.93 ± 0.05 (↑)	33.3	0.0	73.3	0.0	33.3	0.0	73.3	0.0
PPARγ	0.35	0.93 ± 0.02 (↓)	84.0	5.0	87.7	1.3	81.5	2.5	87.7	1.3
PR	0.60	0.53 ± 0.06 (↓)	33.3	0.0	40.7	0.0	22.2	−11.1	40.7	0.0
RXRα	1.0	0.89 ± 0.05 (↑)	35.0	0.0	80.0	0.0	25.0	−10.0	80.0	0.0
COX2	0.80	0.95 ± 0.01 (↑)	65.2	2.6	82.8	−0.2	59.8	−2.8	77.6	−5.4
PDE5	0.85	0.64 ± 0.04 (↓)	31.4	0.0	43.1	3.8	23.5	−7.9	33.3	−5.9

*The NIB model producing the highest EF1%_DEC_ (Table 4) was used in the consensus scoring with PLANTS. When optimal and equal (50/50%) weight is used, all datasets produced better EF1%_DEC_ and EF5%_DEC_ enrichments than the docking*.

a *If the ShaEP weight is 1.0, the consensus score comes entirely from ShaEP rescoring, and, vice versa, if the weight is 0, only the PLANTS score is used. The value of 0.50 corresponds to the situation in which PLANTS docking and ShaEP rescoring effect have equal weight in the results. Both the ShaEP and PLANTS scores were normalized to fit the scale from 0 to 1 before combining them. The consensus scoring was not done to acquire the best AUC enrichment possible and, accordingly, upon a rare occasion the value could decrease (downward arrow) instead improving it (upward arrow)*.

b *ΔEF%_DEC_ corresponds to the EF%_DEC_ difference between the consensus scoring and the original ShaEP rescoring of the same NIB-model*.

**Table 7:** The consensus scoring of the DUD-E datasets.

**Target protein**	**Optimal weight**						**Equal weight**			
	**ShaEP weight**	**AUC**	**EF 1%_DEC_**	**ΔEF1%_DEC_**	**EF5%_DEC_**	**ΔEF5%_DEC_**	**EF1%_DEC_**	**ΔEF1%_DEC_**	**EF5%_DEC_**	**ΔEF5%_*DEC*_**
ER-mixed	0.35	0.69 ± 0.02 (↓)	24.5	6.2	37.9	5.3	23.0	4.7	36.8	4.2
AR	1.0	0.76 ± 0.02 (↑)	13.0	0.0	23.0	0.0	9.3	−3.7	19.0	−4.0
GR	1.0	0.70 ± 0.02 (↑)	5.8	0.0	17.4	0.0	2.3	−3.5	16.7	−0.7
MR	1.0	0.70 ± 0.03 (↑)	11.7	0.0	25.5	0.0	9.6	−2.1	21.3	−4.2
PPARy	0.20	0.85 ± 0.01 (↔)	27.7	17.4	58.1	25.7	21.9	11.2	46.7	14.3
PR	0.55	0.72 ± 0.02 (↑)	6.8	2.4	18.4	1.3	6.8	2.4	18.1	1.3
RXRa	0.25	0.82 ± 0.02 (↑)	19.1	8.4	46.6	22.7	14.5	3.8	29.0	5.1
COX2	0.10	0.69 ± 0.01 (↑)	7.6	5.3	25.5	6.4	6.0	3.7	23.4	4.3
PDE5	0.25	0.82 ± 0.01 (↑)	17.6	7.0	36.4	10.5	13.8	3.2	31.7	5.8
NEU	0.50	0.91 ± 0.02 (↑)	16.3	3.0	52.0	9.1	16.3	3.0	52.0	9.1
CYP3A4	0.50	0.61 ± 0.02 (↔)	10.6	3.0	21.2	2.4	10.6	3.0	21.2	2.4

*The NIB model producing the highest EF1%_DEC_ (Table 5) was used in the consensus scoring with PLANTS. When optimal weight is used, all datasets produced better EF1%_DEC_ and EF5%_DEC_ enrichments than the docking. In the case of equal (50/50%) weight, only the PPARy dataset produced weaker early enrichment than the original docking. See Table 6 for further details*.

This score weighting or consensus scoring (Tables 6, 7) was performed to determine, if the ranking benefitted more from either of the scoring functions and if there is a generally applicable weight ratio that could be routinely used. Because the emphasis in the consensus scoring was put on the EF1%_DEC_ improvement, the AUC values of the DUD datasets were not necessarily improved (e.g., PPARγ; Table 2 vs. Table 6). Similarly, with the ER-mixed, plagued also by the dualistic nature of the included agonist/antagonist ligands, the AUC values were not improved for the DUD-E (Table 3 vs. Table 7). Moreover, focusing on the early enrichment indicates that the consensus scoring worked almost without an exception better than the docking for both the DUD (Table 4 vs. Table 6) and DUD-E datasets (Table 5 vs. Table 7). Even a relatively tiny push by the R-NiB (e.g., 10–35% weight from ShaEP) was enough to help the early enrichment (Tables 6, 7).

Dealing with a completely new target protein cavity or heterogeneous ligand set is likely to require re-weighting and careful optimization upon the arrival of experimental results. Despite this, the yield was in most cases improved by simply giving both scoring functions an equal weight in the consensus scoring (Tables 6, 7) instead of using the default PLANTS scoring or the R-NiB alone (Tables 4, 5). With the DUD datasets, the equal weight consensus scoring produced always better early enrichment than the docking, but the non-weighted R-NiB could sometimes work slightly better (see the negative ΔEF values in Table 6; Figure 2). Similarly, the equal weighting produced better early enrichment than docking scoring alone with the DUD-E datasets; however, the yield for the PPARγ did not benefit from this arrangement. Regardless, with a multitude of targets, the non-weighted R-NiB produced higher early enrichment than the equal weight consensus scoring (see the negative ΔEF values in Table 7; Figure 3).

Although the equal weighting in the consensus scoring could reduce the early enrichment marginally in certain cases, the tradeoff was that in general it produced better early enrichment; making it a viable option for future docking screening experiments.

## Conclusions

This study demonstrates that by simply focusing on the shape/electrostatics complementarity between the ligand and the receptor protein's binding cavity, the docking performance regarding the early enrichment can be improved across the board. The rescoring is done by generating a negative image of the protein's ligand-binding cavity that is then used directly in the similarity comparison of the docking solutions (Figure 1). The results show that the negative image-based rescoring (or the R-NiB) can enhance the success-rate of docking screenings to a level that facilitates effective drug discovery. Moreover, the R-NiB can be used in unison with other docking scoring functions in consensus scoring to improve the early enrichment yet further.

## Author contributions

STK performed the docking and rescoring assays with the assistance from SN and MA. PAP wrote the manuscript with the help from the co-authors. OTP and PAP designed the experiments based on the original concept by OTP and SL. PAP supervised the study.

### Conflict of interest statement

The authors declare that the research was conducted in the absence of any commercial or financial relationships that could be construed as a potential conflict of interest.
